# Context factors for implementation of clinical recommendations for chronic musculoskeletal pain

**DOI:** 10.4102/phcfm.v16i1.4318

**Published:** 2024-09-25

**Authors:** Dawn V. Ernstzen, Quinette A. Louw

**Affiliations:** 1 Division of Physiotherapy, Department of Health and Rehabilitation Sciences, Faculty of Medicine and Health Sciences, Stellenbosch University, Cape Town, South Africa

**Keywords:** chronic musculoskeletal pain, clinical practice recommendations, context factors, implementation, primary health care

## Abstract

**Background:**

Implementing evidence-informed clinical practice recommendations is important for managing chronic musculoskeletal pain (CMSP) to address the multidimensional impact of the condition. Successful implementation of recommendations requires understanding the multiple context factors that influence CMSP management in different settings.

**Aim:**

This study aims to explore contextual factors that could influence the implementation of evidence-informed clinical practice recommendations for the primary health care of adults with CMSP.

**Setting:**

The study focused on the primary health care (PHC) sector in Cape Town, South Africa.

**Methods:**

A qualitative descriptive study was conducted. A multidisciplinary panel of 13 local health care professionals participated in focused group discussions. The participants considered multimodal clinical recommendations derived from published clinical practice guidelines. In four focus group discussions (three or four members per group), the panel generated and documented context factors that would influence implementing the recommendations in practice. Inductive content analysis was performed to identify categories and themes.

**Results:**

The five contextual themes generated indicated health care system organisation, human resource requirements, provider practice patterns, patient empowerment and integration into policy as imperative for the successful implementation of recommendations.

**Conclusion:**

There are diverse context factors that could influence the implementation of clinical recommendations for managing CMSP in PHC settings. Identifying these factors as barriers or facilitators is beneficial for developing effective knowledge translation strategies.

**Contribution:**

The study findings indicate that an integrated systems approach supported by health care policy and multisectoral collaboration is needed to successfully implement clinical recommendations to address the impact of CMSP.

## Introduction

Chronic pain is described as pain that has persisted for more than 3 months and thus beyond expected healing time.^1,2^ Globally, about one in five people suffer from chronic pain.^2,3^ In South Africa (SA), 20% of women and 18% of men reported chronic pain, and the prevalence of chronic pain increased with age.^3^ The International Classification of Diseases, 11th edition (ICD-11) classifies chronic pain as chronic primary pain, chronic cancer pain, chronic post-surgical pain, chronic neuropathic pain, chronic headache and orofacial pain, chronic visceral pain and chronic musculoskeletal pain (CMSP).^2^ Chronic musculoskeletal pain is described as persistent or recurrent pain that is associated with bone, joints, muscles and soft tissue involvement. Musculoskeletal conditions are one of the top contributors to disability globally and in sub-Saharan Africa.^4,5^

The first point of entry for CMSP care is primary health care (PHC). It is, therefore, imperative to optimise CMSP management in PHC to address the health, economic, health care system and social impact of the condition. Implementation and uptake of evidence-informed clinical recommendations from clinical practice guidelines (CPGs) can optimise the quality of care and are foundational for robust health care systems.^6^ In low- and middle-income countries (LMICs), the optimisation of care is often hampered by factors such as a lack of clinical guidance. Therefore, there is a drive to develop evidence-based recommendations that are appropriate for the local context. However, the implementation of such recommendations in clinical practice remains a challenging task and is influenced by multiple contextual factors.

Published evidence from three systematic reviews provides insight on factors that influence the implementation of clinical recommendations for musculoskeletal disorders (low back pain, neck pain and CMSP).^7,8,9^ These factors comprise the identification of context-specific implementation barriers and facilitators, patient engagement, sustainable implementation and theory-driven intervention strategies, which may improve the uptake of clinical recommendations.^7,8,9^ The reviews provided little insight into the implementation strategies in LMICs. Low- and middle-income countries present many additional challenges to implementation, such as resource constraints and a lack of local, contextually relevant evidence.^10^ More information is required regarding the factors that may impact implementation strategies for CMSP care in LMIC contexts.

**FIGURE 1:**
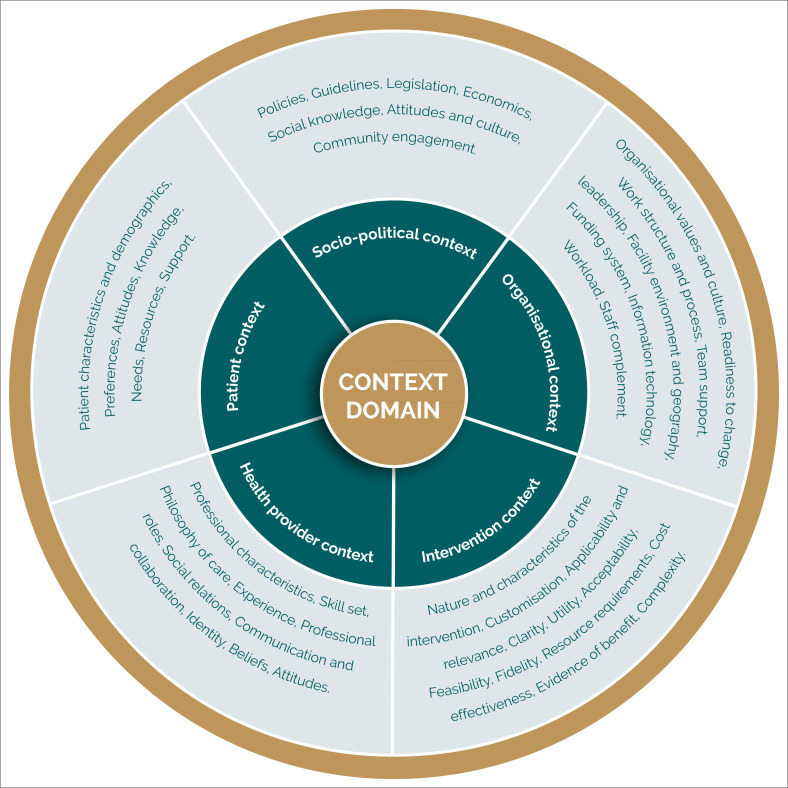
Summary of context domains, context attributes or features that influence the implementation of evidence in practice.^12,13,14,15^

Context factors may hinder the uptake of evidence-based interventions despite efforts to implement them, thereby preventing meaningful patient and health care system outcomes.^11^ Context is described as the characteristics or circumstances of a setting in which evidence is to be implemented.^11,12,13,14,15^ Various published studies examined domains, attributes and characteristics of context for the implementation of evidence. For example, Squires et al.^13^ extracted 16 context attributes with 66 different context factors from 11 studies. Figure 1 summarises different context domains, with examples of context attributes and factors in a conceptual framework. The generic context domains comprise the socio-political context, the health care organisation context, the context of the health professionals, the patient–client context, the characteristics of the intervention and its process of implementation. The knowledge base on defining the various aspects of context has developed considerably as context factors are instrumental in explaining the mechanisms by which interventions work and the relevant environments and contexts.^16,17^

Contextual consideration is essential for knowledge translation in health care, because context factors may act as barriers or facilitators to implementation. There is a need to systematically study the attributes of context that may influence the feasibility, acceptability and implementation of interventions.^16,17^ Understanding the context in a setting can address barriers to implementation and optimise the effectiveness of implementation strategies. Context factors inform the tailoring of interventions and implementing strategies to enhance acceptability in the local setting. Hence, the ‘fit’ between the intervention and the context is critical in determining the success of implementation.^6,15^

Information regarding translating evidence into practice for CMSP in the South African context will be valuable to address the impact of the condition. Recently, a set of clinical practice recommendations for PHC of CMSP in SA has been developed. These recommendations were developed through a review of high-quality CPGs relevant for PHC, and the recommendations were endorsed for the South African context by a multidisciplinary expert panel.^18,19^ Evidence-informed guidance in SA can play an important role in the rollout of policies such as the National Health Insurance (NHI) of South Africa,^20^ which aims to provide equitable and cost-effective health care. This study explores the context factors that may influence the implementation of evidence-informed multimodal clinical recommendations for the PHC of adults with CMSP in Cape Town, SA. Such information may contribute to developing a tailored plan to implement clinical recommendations.

## Research methods and design

### Study design

A qualitative approach with a descriptive exploratory design^21^ was used to explore and describe factors that could influence the implementation of evidence-informed clinical practice recommendations for the PHC of adults with CMSP in Cape Town, SA. The study formed part of a larger project focused on developing evidence-informed clinical practice guidance for the PHC of CMSP. The initial study phase focused on summarising and synthesising evidence-informed clinical practice recommendations for CMSP through a systematic review.^19,22^ Thereafter, the clinical recommendations were contextualised and endorsed for use in a South African context by a multidisciplinary panel.^18^ This study focused on factors that may influence the implementation of the recommendations.

### Setting

This study focused on the public sector PHC settings in Cape Town, SA, which serve as the entry point for patients into the care continuum and also for patients with CMSP. Primary health care in SA comprises home and community-based care and primary care services delivered at community health care centres and community clinics.^23^ The services provided include preventive, promotive, curative, rehabilitative and palliative interventions.^23,24^ The diagnosis and holistic management of CMSP can be provided at the PHC level; however, when treatment goals are not achieved at the PHC level, the patient may be referred to pain services at higher levels of care.^18,23^

### Sampling of participants

A multidisciplinary local group of 17 health care professionals was identified through purposive sampling and invited to participate. The sample size was based on the principle that four focus groups of four members would be sufficient for code saturation in this study with a distinct aim.^25^ The sample size and the sampling criteria comprised skills and experience with CMSP management; experience in CPG writing and use; working or have worked in the local health care environment; a range of diverse health care professionals involved in pain care, namely, medical doctors, clinical nurse practitioners, physiotherapists, occupational therapists, pharmacologists, psychologists, managers and researchers; and having at least 3 years’ experience in the field. The research team identified potential participants through their contributions to conferences focusing on pain management and public health, publications on the topic and public health care sector involvement. These potential participants were then requested to suggest other potential participants who may fit the inclusion criteria.^26^ The principal investigator (PI) invited potential participants and explained the study purpose and procedures via email and/or personal discussion.

### Instrumentation

Four in-person focused group discussions (FGDs) were conducted, allowing interactive discussion between participants about key issues that would be relevant for implementing clinical recommendations. Box 1 summarises the discussion task provided. One member documented key points from the discussions on a custom-built discussion guide, which was designed by the research team based on a similar study (Appendix 1).^27^

BOX 1 Defining the task for the focused group discussions.

**Table:** 

**Activity prior to the group discussion** Overview of the clinical practice recommendations.	
**Purpose of the discussion:** To generate information on factors that may impact the acceptability, feasibility and implementation of the evidence-informed practice recommendations for primary health care of chronic musculoskeletal pain in the South African context.	
**Task:** Please refer to the clinical recommendations that have been assigned to your group. Discuss any locally relevant aspects that might influence the acceptability, feasibility and implementation of the above-mentioned recommendations in the South African public primary health care sector. You may list any context points or practice points that may influence the implementation of the recommendations in the local context. Please use the discussion guide to inform the process of identification of context and practice factors and document your discussion points on the discussion guide provided.	
**Key terms:**	
Acceptability: Feasibility: Implementation: Context points: Practice points:	Appropriateness for the context. Practical to apply in the context. Ability to be included in routine practice. Personal, environmental and resource factors in a setting that could influence implementation strategies. Practical issues that may support or hinder the implementation of recommendations in the South African context.

### Procedures

The FGDs took place in Cape Town, SA, within 1 day. All attendees provided written informed consent before the meeting. Prior to the FGDs, as part of the Delphi study18 the participants were presented with the clinical recommendations, accompanied by the strength of the body of evidence for each recommendation (Table 1). The participants were thus familiar with all clinical recommendations and the evidence underpinning them prior to the FGDs. Table 1 provides a summary of the clinical recommendation topics.

**TABLE 1:** Key topics covered in the core list of clinical recommendations.

Group number	Topic	Focus of the clinical recommendations
1. Approach to care and assessment	Approach to care	Patient-centredness; shared decision-making and goal setting; inter-professional collaboration
	Assessment	Holistic assessment; assessment instruments; reassessment; classification of pain; diagnostic and imaging procedures
2. Non-pharmacological management	Physical therapy	Manual therapy; exercise therapy; adherence to exercise
	Electrotherapy	Transcutaneous electrical nerve stimulation
	Psychological therapy	Identify mental health needs; refer to a psychologist; and use operant behavioural therapies, cognitive behavioural therapies and respondent behavioural therapies
	Complementary medicine	Acupuncture
3. Pharmacological management and further referral	Pharmacological management	Analgesic review; paracetamol; NSAIDs and risk assessment; antidepressant therapy; antidepressant therapy review; anti-epilepsy or anti-convulsants; opioid therapy with risk assessment and informed consent
	Multidisciplinary management	Multidisciplinary management team
	Specialist referral	Pain management specialist
4. Education and self-management	Advice and education	Address concerns; advice to stay active; brief education; education about analgesia; pain neuroscience education
	Self-management	Supported self-management

*Source:* Ernstzen DV, Parker R, Ras T, Von Pressentin K, Louw QA. Clinical recommendations for chronic musculoskeletal pain in South African primary health care. Afr J Prm Health Care Fam Med. 2023;15(1):a3929. https://doi.org/10.4102/phcfm.v15i1.3929, for more information

The participants worked in focused groups to complete the task (Box 1). Each group focused on a particular topic, namely: (1) approach to care and assessment, (2) non-pharmacological management, (3) pharmacological management and further referral, and (4) education and self-management. The group allocation was flexible and was determined on the day of the meeting based on participants’ expertise and preference. Each FGD was audiotaped for audit purposes (four different recordings). The PI chaired the meeting, explained the task and answered any questions regarding the recommendations or the FGD process.

### Data management and analysis

The written documents containing the context information were collected from the groups and typed verbatim by the PI to produce four MS Word files (one per group). Where content uncertainty existed, the PI verified the information using the audio recording of the FGDs.

The data analysis focused on contextual factors that were generic across the focus groups, using inductive content analysis.^21^ The PI organised the data based on content similarity. Thereafter, the PI compared the information and extracted the common categories.^28^ The categories were then reorganised into themes. The themes were deductively matched to the contextual domains within the conceptual framework in Figure 1. The PI conducted the initial data analysis, which was further developed by a research team member and a research assistant. The research team explored the relationships between categories and between themes. The research team members were physiotherapists with training and experience in qualitative research methods and CPG development. Two research team members had experience in working in PHC in SA, while the research assistant was from the Philippines.

### Trustworthiness of qualitative data

Credibility was enhanced using source triangulation by comparing the data generated in four groups, discussing different clinical recommendations and consisting of diverse members. The above example of data source triangulation seeks convergence and corroboration of information.^29^ Additionally, member checking was done to assist with data validation by e-mailing a summary table of context factors to selected participants. Iterative data analysis between the data from the different focus groups was used to facilitate dependability. Furthermore, the data collected were sufficient to answer the research aim and indicated congruence with the conceptual framework of contextual domains (Figure 1), although more data collection opportunities may have provided additional information. To aid confirmability, a research assistant and a research team member audited the summary of the documented information for peer debriefing. For transferability, a description of the context and sampling strategy has been provided, and a framework on contextual factors for implementation was used in the data analysis and interpretation.^29^

### Ethical considerations

The study protocol was approved by the Stellenbosch University, Health Research Ethics Committee (HREC) (Protocol number: S14/01/018). Written informed consent was obtained at the meeting. The participants also had to declare any personal, professional or financial conflicts of interest (none was noted). Because of the nature of the in-person focus groups, participants’ contributions were known to each other; therefore, participants committed to upholding the confidentiality of information shared in FGDs. The documents from the FGDs did not contain any participant identifying information. Participants were aware that they could exit the study at any point in time.

## Results

### Demographic information of participants

Of the 17 potential participants who were invited to participate, 13 participated in the FGDs (three or four members per FGD) (see Table 2). Travel distance and work commitments were the main reasons that limited participation in the study. Physiotherapists were the largest professional group present in the FGDs.

**TABLE 2:** Demographics of the participants.

Demographics	(*N* = 13)	%
**Participants**		
Women	12	92
Men	1	8
**Occupation**		
Clinical nurse practitioner	1	8
Medical doctor	1	8
Occupational therapist	1	8
Physiotherapist	8	61
Manager: pain clinic	1	8
Social anthropologist	1	8

Five themes emerged from the data, indicating context factors that may influence the applicability, feasibility and implementability of clinical recommendations. These five themes comprised the integration of the recommendations with policy and existing guidelines, the need for health systems strengthening, human resources to deliver evidence-informed interventions, practice patterns that will enable implementation (including training) and contextually relevant patient education. The five themes are elaborated on, and quotes extracted from the documented information are provided in Table 3. Quotations are identified from the FGDs (e.g. FGD 1 indicates a quote from focus group 1).

**TABLE 3:** Themes of context factors for the implementation of the clinical recommendations for primary health care of chronic musculoskeletal pain in South African settings.

Theme	Category (Context factor)	Code (Context or practice point)	Quote
**DOMAIN: Societal and policy context**			
**THEME 1: Integration into policy and existing guidelines**	**Policy Integration**	Healthcare 2030 Social welfare policies Mental health policy	*Integrate as part of CCAIRR (Healthcare 2030). (FGD 1)* *Include in EML; however, include rehabilitation options in EML; Certain medicines are not listed on the EML for PHC SA and are thus not available in this context and cannot be prescribed; Integrate approach as part of PACK; Create linkages with Social Services; and Refer to Department of Social Services if social determinants affect health. (FGD 2)* *Exercise as a core part of wellness must appear in policies, for example, the Western Cape on Wellness project; Integrate into National Policy (look at mental health policy). (FGD 3)*
	**Available guidelines**	Standard treatment guidelines Practical Approach to Care Kit	
	**Community engagement**	WoW initiative	
**DOMAIN: Organisational context**			
**THEME 2: Health system organisation**	**Availability of multimodal care**	Availability to an interdisciplinary team Availability of medication or analgesics	*A combination of pharmacological and non-pharmacological management is needed to optimise the treatment of depression in chronic pain (FGD 3); Access to non-pharmacologic management options (rehabilitation) professionals; Access to an adequate amount of medication. (FGD 2)*
	**Interdisciplinary team required**	Prescribing clinicians (medical doctor or clinical nurse practitioner) Chronic care nurse Dispensing clinicians (pharmacist) Rehabilitation clinicians (physiotherapist, occupational therapist) Mental health practitioners (psychologist, psychiatric nurse, psychiatrist) Social worker Community workers or volunteers may play an important role in community-based initiatives	*Interdisciplinary team (required): doctor, physiotherapist, occupational therapist, psychologist, nurse and social worker, occupational health nurse, dispensing clinicians (FGD 2), chronic care nurse (FGD 3) and occupational therapy for work interventions. (FGD 4)* *However, holistic assessment is important at the first contact clinician, which can be the nurse, medical officer or, in some cases, the physiotherapist. (FGD 1)* *For multidisciplinary care, Lead can be a nurse, physio- or occupational therapist. (FGD 2)* *Access to a physiotherapist (not all centres have physiotherapists and some have only sessional physiotherapists). More physiotherapists are needed on the platform. (FGD 3)* *Psychologists are not readily available in this context and may need to seek alternatives. (FGD 3)* *Mental health practitioner (psychologist, psychiatric sister, social worker, psychiatrist) required; Access to clinicians trained in cognitive behavioural therapy. (FGD 3)* *A social worker and psychologist should be included. (FGD 1)* *Physiotherapists can train assistants of volunteers to present exercise classes in the community. (FGD 3)* *Support programmes in the community can be led by a nurse, physiotherapist, occupational therapist, trained assistants or community workers. (FGD 3)*
	**Work structure**	Collaboration Interdisciplinary team members Streamlined referral process Referral guidelines to facilitate appropriate referral	*Access to specialists is challenging in this context. There is a need to shorten waiting times and streamline referral systems for better access. This increases the need for interdisciplinary collaboration and a good referral system in the clinic. (FGD 2); Where needed, consult with a pain specialist; If pain persists, refer to a tertiary centre (pain clinic). (FGD 2)* *No multidisciplinary pain programmes are available in the PHC context. Therefore, there is an urgent need for interdisciplinary collaboration in clinics (within the facility). (FGD 2)* *Guidelines on when to refer (within competency and scope of practice) needed. (FGD 1)*
	**Communication system**	Electronic communication system Regular team meetings Regular feedback to colleagues	*The interdisciplinary team should have regular meetings to enhance communication, collaboration, contact and early referral. An electronic communication system is essential for providing feedback to the team. (FGD 1)* *Clear referral lines and accessibility to pain clinic; Appropriate pathway of care important. (FGD 2)*
	**Health care system capacity**	Adequate consultation time per patient Measures for continuity of care	*To enable a holistic approach, more time per patient and an interdisciplinary team are required; the patient should be re-assessed at follow-up by the same treating clinician to enhance continuity of care. (FGD 1); A solution to the system overload is imperative. (FGD 1)* *Will need enough time with the patient at first visit; thus, more time per patient is required. (FGD 3)*
	**Resources**	Infrastructure and equipment	*Room, outcome measures, equipment, patient educational material. (FGD 2)* *Gym space to perform individual or group exercises in a safe area. (FGD 3)*
**DOMAIN: Intervention implementation context**			
**THEME 3: Practice patterns or work structure**	**Holistic assessment**	Good communication skills, patient-centredness; and cultural sensitivity Changes in social environment or symptoms Classify pain	*Applying patient centredness is important at the first visit to build rapport and gain the patient’s trust; Cultural appropriateness will enhance the approach; Communication skills, such as appropriate language and appropriate information at the level of the patient. (FGD 1)* *Check for change in social environment or physical symptoms. (FGD 1)* *Important to identify the type of pain – may also use a diagnostic tool. (FGD 1)* *Assess for the type of pain (inflammatory vs mechanical vs neuropathic) to prescribe accordingly. (FGD 2)*
	**Outcome measure use**	Outcomes-based approach Appropriate screening tools and outcome measures	*Simple, short and validated screening tools and outcome measures will ease holistic assessment and interdisciplinary communication; One holistic tool relevant to the context would be ideal. (FGD 1)* *Use a short, validated questionnaire to identify psychological distress. (FGD 2)* *Screening tools available in different languages and culturally relevant. (FGD 3)*
	**Modes of interventions**	Group intervention Patient education Work-based interventions Supported self-management Community-based support programme Diverse educational material	*Establish a chronic pain management programme in various clinics and discharge to a community club. (FGD 2)* *Can be delivered through a group exercise programme. This is important to accommodate the volume or number of patients in primary health care. (FGD 3)* *For continuity of care and adherence to exercise; A chronic pain club should be established at the clinic and in the community. (FGD 3)* *Access to the work sector to deliver education and advice on occupational health is needed. (FGD 4)* *Audio-visual material, e.g., educational videos in waiting areas; Refer to trustworthy self-help and audio-visual material. (FGD 4)* *Must have educational leaflet, decision-making aid, poster, multimedia. (FGD 2)*
	**Risk management**	Risk assessment strategies A risk management system for adverse effects Safety considerations for exercise and equipment Adherence to prescribed interventions Use of home remedies or over-the-counter medicines	*Risk management when patients present with side effects to ensure continuity of care system. (FGD 2)* *Use of the WHO Step care or ladder approach is important. Thorough assessment to identify indications, co-morbidities, risks, and precautions (risk assessment). (FGD 2)* *Check simultaneous use of home remedies or over-the-counter medicines. Check for side effects and adherence to prescribed medication. (FGD 2)* *A thorough assessment by the physiotherapist to identify risks of precautions for exercise. Develop an individualised exercise programme; cost and safety with electrotherapy application must be considered. (FGD 3)*
**DOMAIN: Health provider context**			
**THEME 4: Capacity Building**	**Skills development**	Diverse training needs; which may include: ■ Motivational interviewing ■ Group interventions ■ Health promotion ■ Occupational health ■ Pain neuroscience education ■ Cognitive behavioural therapy ■ Risk screening ■ Clinical guidelines use	*Skills training may be required to use the approach, such as communication skills training and chronic pain management. (FGD 1)* *Communication skills training may be required (e.g., motivational interviewing) (FGD 3); Patient centredness and communication skills are important. (FGD 2)* *Training within the scope of practice of prescribing or dispensing clinician (FGD 2); Guidelines on when to refer (within the competency and scope of practice). (FGD 1)* *Training according to the type of exercise delivered and education in exercise physiology; training for identification and management of depression; may need training in cognitive behavioural therapy; relaxation therapy training. (FGD 3)* *Staff may initially need training to use assessment tools. (FGD 2)*
**DOMAIN: Patient context**			
**THEME 5: Contextually relevant education**	**Patient and caregiver empowerment**	Education for understanding and adherence Family education Education in the workplace	*Understanding will enhance adherence; the family needs to be involved in training. (FGD 3)* *Family education may enhance support. (FGD 1)* *Educate employers and colleagues at the workplace about the condition. (FGD 4)*
	**Participation in care**	Patient empowered for shared decision-making Include diverse educational topics to aid understanding and improve adherence Teach skills for self-management	*Patient education about the cause of pain and the need for referral; patient understanding important; patient education about benefits, limitations and risks of imaging. (FGD 1)* *Patient education on the role of medication in chronic pain management. (FGD 2)* *Education – about chronic pain and depression; Teach patient relaxation skills to do at home; Teach patients home or self-mobilisation and exercise to maintain the effects of manual therapy; Teach coping skills. (FGD 3)* *Educate patient and family about the benefits of staying active and PNE. (FGD 4)*
	**Person-centred care**	Consider patient preference Education or explanation using appropriate language	*Explain findings to the patient using appropriate language; the patient should be empowered to take part in decision-making. (FGD 1)* *The prescribed exercise must be specific to the condition and diagnosis and consider cultural aspects. (FGD 3)* *Consider patient preferences, E-resources, for example, self-help sites for patients. (FGD 3)* *Patient educational material (printed) in different languages is very important; PNE applicable stories in different languages are needed, ideally validated for the South African context. (FGD 4)*

CCAIRR, Caring, Competence, Accountability, Integrity, Respect & Responsiveness; EML, essential medicines list; FGD, focus group discussion; PACK, Practical Approach to Care Kit; WoW, Western Cape on Wellness; PHC SA, Primary Health Care, South Africa; WHO, World Health Organization.

### Theme 1: Integration with policy and existing guidelines

The integration of recommendations in policy and other South African guidance documents was named as a facilitator for implementation. Several policies and existing health guidelines were listed as examples, namely, Healthcare 2030,^24^ the standard treatment guidelines (STG) for PHC in SA,^30^ PACK (Practical Approach to Care Kit),^31^ the WoW (Western Cape on Wellness)^32^ initiative, mental health policy and the social welfare policy.

### Theme 2: Health system organisation

Theme 2 was the largest and focused on the importance of health services organisation at PHC for implementation. An essential prerequisite for the implementation of the clinical recommendations was access to multimodal care and the availability of the necessary workforce. Participants explored the roles of nurses as first-line practitioners, medical officers for prescribing, physiotherapists for physical rehabilitation interventions, mental health practitioners for mental well-being and the role of community health workers (CHWs). Physiotherapists and mental health practitioners were seen as a core part of the chronic pain management team, but their availability in PHC was restricted. In addition to clinicians, community members were seen as integral to community-based interventions and adherence to chronic pain care. The participants were concerned about the PHC system’s capacity to implement the clinical recommendations. The system load resulting in insufficient consulting time per patient was classified as a barrier to interdisciplinary care and continuity of care. Efficient interdisciplinary collaboration between team members was the next important consideration to facilitate efficient communication. Streamlined communication and referral procedures were nominated as a key factor in the implementation of recommendations to positively influence continuity of care and timely access to intervention options.

### Theme 3: Capacity building

Participants suggested that health care provider training could be required to build capacity for seamless implementation of the clinical recommendations. A variety of training needs were listed, and the scope of practice played a role in training needs. Communication training was considered important for a holistic approach to care and to provide person-centred care.

### Theme 4: Practice patterns that will enable implementation

Participants elaborated on practice patterns that possibly needed to be refined and strengthened. They focused on holistic, person-centred and culturally appropriate assessment as a cornerstone in planning appropriate chronic pain management. Such assessment would initiate and direct an efficient patient care pathway via optimised communication skills. An outcomes-based approach, using validated and contextually appropriate outcome measures, was emphasised for the efficiency of assessment. Guidance for appropriate referral and risk management were also key strategies to be reinforced. Specific aspects were methods to screen for and educate about side effects from medication and the interaction between prescribed and non-prescription medication. Evaluation of risks associated with exercise and electrotherapy interventions were also considered. Although a group intervention was not a specific clinical recommendation, participants thought many of the clinical recommendations could be delivered in a group-based setting, followed by participation in community support groups for sustainability.

### Theme 5: Contextually relevant education

Educational interventions, such as family education and workplace-based educational interventions, were seen as foundational to empower these stakeholders with the knowledge to best support the person with chronic pain. The participants highlighted various education topics, including explanations of assessment findings, risks and benefits of interventions and specific skills development. Participants expressed that for education to be taken up, it must be contextually relevant and person-centred. There was a preference for printed educational material, although electronic sources were mentioned.

## Discussion

This study found several multidomain context factors that may influence the acceptability, feasibility and implementation of clinical recommendations in the PHC sector for CMSP in Cape Town, SA. Five main themes summarised the context factors that may influence the successful implementation of the clinical recommendations. The contextual themes comprise the integration of recommendations into policy and guidelines, the capabilities of the health care system, sufficient human resource capacity, streamlining practice patterns and considering patient context. The context factors identified fit within the contextual domains framework (Figure 1) but relate specifically to CMSP care in the local, Cape Town context. Our study addresses the knowledge gap regarding understanding health care system responses to the burden of musculoskeletal conditions in LMICs.^33^

A robust health care system is required to address the significant burden of pain and disability from musculoskeletal conditions in LMICs. Strengthening of health systems in LMIC settings is constrained by the socio-political context and not acknowledging musculoskeletal conditions as a non-communicable disease (NCD) in health care policies and guidelines.^33^ Congruently, existing CPGs for SA focus on the quadruple burden of disease, and no guidelines for musculoskeletal health exist.^34^ It is therefore advocated that guidance for musculoskeletal health be integrated into public health initiatives and the management of NCDs.^33^ Participants acknowledged several policies into which evidence-informed recommendations can be integrated. However, a National Pain Strategy is an important consideration to coordinate care and address the impact of pain in communities.^35^ In SA, with the establishment of the NHI, there is an opportunity to prioritise musculoskeletal conditions because it is the fifth top contributor to Years lived with disability in SA^5^ and has the highest need for rehabilitation globally.^4^ Efforts have been made to address the gap in prioritisation by including chronic pain care in PACK^31^ and including rehabilitation recommendations into the STGs for PHC.^30,36^ Multisectoral policy integration and access to the rehabilitation team are needed towards health system strengthening and addressing the burden of pain and disability.

The World Health Organization’s (WHO) six building blocks for health system strengthening comprise service delivery, health workforce, health information systems, access to essential medicines, financing, and leadership or governance.^33^ Our findings highlight components of these building blocks for health care system strengthening for CMSP in the local PHC context, namely, efficient referral pathways, availability of an interdisciplinary team, skillset of the team, efficient communication, infrastructure, availability of analgesics and policy to practice factors. Although our study did not focus on identifying enablers and barriers but rather on factors that could affect implementation, several implementation challenges were identified. These challenges were congruent with published challenges for public health care in SA^37^ and globally.^33,38,39^ The challenges to implementation were human resource limitations and the time available to consult with patients, which are linked to the high demand for services care at PHC centres.^37^ Additional limitations, such as lacking electronic records, could influence communication between team members. The findings of interdisciplinary communication restraints indicate the need for a model of care to assist in navigating the current health care system. However, before a model of care for CMSP can be implemented, the health care system’s readiness to change needs to be ensured, not to overburden an already strained system.

To achieve the goal of interdisciplinary care, a range of health care practitioners with the required skill set to implement the recommendations is ideal. The lack of access to rehabilitation practitioners and mental health practitioners was noted as a concern in the local context. The study findings provide several suggestions regarding training staff to build a skill set for implementation, such as training for holistic assessment, outcome measures and risk management strategies. Globally, health care providers face similar challenges in translating knowledge into clinical practice because of the lack of reliable and validated measures for pain, concerns about adverse effects and limitations in patient contact time.^40^ In addition to providing training, innovative ways to enable interdisciplinary care must be developed to adapt a workflow suited for the local context, which could involve task shifting. Planned and structured opportunities to deliver interventions and to regularly communicate regarding patients’ progress are a further implementation consideration.

A group-based intervention can potentially address some of the barriers to implementation. There is growing evidence that group interventions for CMSP are beneficial to address pain and disability, to provide social support and to empower patients.^41,42,43^ The suggestion of an interdisciplinary group intervention for patients with CMSP, followed by a support group in community settings, could be a relevant solution to the current restrictions on the availability of the interdisciplinary team. Furthermore, the involvement of a CHW in facilitating support groups in communities may optimise sustainability and adherence in CMSP management, which is promising.^44,45^ The contribution of the CHW as part of the SA PHC workforce and in connecting communities with health care services is widely acknowledged.^45,46^ Adequate rehabilitation workforce size, training and funding are needed to address the increasing need for rehabilitation in the local public health sector to optimise functioning, quality of life and return to productive work for people with CMSP.^5^

Contextual relevance of assessment and interventions was consistent throughout the emergent themes. The findings indicate a need for assessment instruments and educational and clinical interventions to be suited to the needs and resources of the patient. Contextual relevance, therefore, implies a person-centred approach to management, which considers the patient’s knowledge and beliefs about pain, how they cope with pain, family and work factors that impact pain, and the patient’s goals and expectations to tailor interventions accordingly.^47^ Strategies to facilitate person-centred care from our study comprised adequate consultation times, relevant educational material, family involvement and return to work strategies. Additionally, peer-led and CHW-led pain programmes delivered in the community may be instrumental in tailoring interventions to be contextually relevant and culturally sensitive.^48,49^ Community health workers are proposed to have an understanding of the community culture and language to be able to deliver tailored interventions, which may enhance the uptake of the recommendations by patients to achieve the end goal of supported self-management.^50^ The above may contribute to the outcome of an empowered patient, which could positively influence health-seeking behaviour and the high demand for services at PHC.

### Strengths and limitations

The study findings provide foundational information on context factors that may influence the implementation of clinical recommendations for the PHC of CMSP in Cape Town, SA. Successful implementation requires a capacitated workforce and sufficient human resources to enable access to an interdisciplinary team. This efficient health care organisation includes community involvement and attention to patient-specific needs. The above requirements would need to be supported by health care policy and multisectoral integration. Contextually relevant models of care can play a role in health care reform, strengthening health care systems and operationalising evidence-based practice.^10,39,51^ Our study identified barriers to implementation. Addressing these barriers and capitalising on facilitators may play a role in narrowing the known evidence-to-practice gap and the musculoskeletal burden-to-response gap.^33^ As context factors that affect implementation are diverse and may differ across settings^15^, we recommend that PHC centres conduct an evaluation of barriers and facilitators to implementation using a context assessment instrument.^12,17^ A thorough understanding of context can enable effective knowledge translation strategies and promote the uptake of recommendations.

Our investigation was specific to CMSP and to Cape Town, SA, and may not be generalisable to other chronic conditions, other LMICs or other parts of SA. Because of the iterative nature of the larger project, the participants only participated in one focus group on a specific topic, which may have limited the depth and breadth of the discussion. The sample size was small, and the participants self-selected the group they preferred to participate in, which may have influenced the content of the discussions. A follow-up meeting is recommended for deeper analysis of contextual issues and confirmation of data saturation. We acknowledge that because of the study’s exploratory nature, we did not study the full scope of acceptability, feasibility and implementability of health care interventions.^14^ Our FGDs were in-person only, and we recommend that future discussions on the topic should include an online component for a hybrid approach to optimise representation. The use of a hybrid mode may optimise participation from participants from different geographical areas and a balance between rural and urban representatives. Although a multidisciplinary group of health care practitioners were invited to participate, those who participated were mostly physiotherapists, and we could not recruit a psychologist and a pharmacist to attend. The panel composition may have influenced the range of findings and was probably influenced by the characteristics of the research team (being physiotherapists). Another shortcoming of the study is that patients and/or families were not represented on the panel. Implementation endeavours to improve patient outcomes must also consider the patient’s needs and resources.^11^ The participation of patients and their families may have elicited more information on cultural and contextual realities. However, we declare that patients and potential end-users provided input on context factors as part of the larger project.^52^

## Conclusion

Several multidomain context factors that could play a role in successfully implementing evidence-informed clinical recommendations for CMSP in a transforming health care system were identified. These contextual factors could act as barriers or facilitators to the uptake of evidence for CMSP. Our investigation was limited to Cape Town, SA. Further investigations should include more diverse multidisciplinary participants from different geographical regions to investigate contextual factors relevant to the broader South African context.

## Appendix 1

**TABLE 1-A1:** Worksheet example.

We endorse performing a complete and holistic patient evaluation which includes history, physical examination, functional status, psychosocial risk factors and contextual factors in evaluating, diagnosing and managing patients with CMSP.
Evidence base: There is evidence
**PHC Contextual factors: contextual factors that may affect the implementation of this recommendation**
**What is needed to implement this intervention?**
Organisational
Practice method (how)
Staff
Resources
Training
Timing (when)
Re-assessment
Referral
Consumer or patient or family member
Policy
Other?

CMSP, chronic musculoskeletal pain; PHC, primary healthcare.
